# Development and First Phase Evaluation of a Maternity Leave Educational Tool for Pregnant, Working Women in California

**DOI:** 10.1371/journal.pone.0129472

**Published:** 2015-06-24

**Authors:** Elaine Kurtovich, Sylvia Guendelman, Linda Neuhauser, Dana Edelman, Maura Georges, Peyton Mason-Marti

**Affiliations:** 1 School of Public Health, University of California, Berkeley, California, United States of America; 2 March of Dimes, California Chapter, San Francisco, California, United States of America; Örebro University, SWEDEN

## Abstract

**Background:**

Despite the provision of maternity leave offered to mothers, many American women fail to take leave.

**Methods:**

We developed an evidence-based maternity leave educational tool for working women in California using participatory design. We tested its short-term efficacy with a randomized controlled trial of pregnant English-speakers (n=155).

**Results:**

Among intervention participants exposed to the tool, 65% reported that they learned something new; 38% were motivated to seek more information; and 49% said it helped them plan their maternity leave. Among participants who delivered at ≥ 37 weeks gestation and said the tool helped them plan their leave, 89% took more than one week of prenatal leave, a significantly higher proportion than among controls who did not receive the tool (64%, p=0.049). Other findings favored trial participants, but were not statistically significant in this small sample. More intervention participants took some prenatal leave (80%) vs. controls (74%, p=0.44). Among participants who had returned to work when surveyed (n=50), mean postnatal leave uptake was on average 1 week longer for intervention participants vs. controls (13.3 vs. 12.2 weeks, p=0.54).

**Conclusions:**

The first-phase evaluation of this tool shows that it successfully informed women about maternity leave options, clarified complex regulations, encouraged women to seek further information and helped plan maternity leave. Compared to controls, trial participants who used the tool to plan their leave were far more likely to take prenatal leave close to term. Future evaluation of the tool when mediated by a health provider or employer is warranted.

## Introduction

Research increasingly shows that mothers who take maternity leave can improve perinatal health outcomes for themselves and for their newborns. Prenatal leave taken routinely in uncomplicated pregnancies may protect against obstetric complications during labor and delivery, low birth weight and small for gestational age infants. [1–6] Maternity leave taken in the ninth month of pregnancy has been associated with a reduced risk of a primary cesarean section. [4] Similarly, delaying a return to work for at least 12 weeks postpartum has been shown to be associated with more timely well baby visits and infant immunization schedules, [7] longer duration of breastfeeding [8–10] and with lower rates of postpartum depression and maternal depressive symptoms. [11,12]

Despite the importance of maternity leave, many American women fail to take adequate advantage of this option. Nationally, most pregnant mothers work full time into their last month and about 40% return to work within three months after giving birth. [13] This amount of leave is short compared to that taken by women in most of the developed world. [14] This is not surprising because the United States (US) is the only industrialized country that does not offer paid leave. [15] The 1993 Family and Medical Leave Act (FMLA) grants workers who meet strict eligibility requirements 12 weeks of unpaid, job protected leave, during which time they can prepare for, deliver and care for the newborn. In contrast, paid leave benefits in all other industrialized countries range from 13 weeks in Iceland to 45 weeks in Norway. [16]. Given the lack of a universal maternity leave entitlement and wide variability of state and employer provisions, many American mothers lack information and support to make maternity leave decisions. Maternity leave requires weighing potential advantages and disadvantages of leave-taking and choosing between alternative courses of action for which there is no right or wrong answer. As a result, decision-making may pose a dilemma or conflict. [17] Inadequate knowledge, unrealistic expectations about leave, unclear values, social pressure, inexperience with negotiating in the workplace, and lack of financial and childcare resources may also contribute to decisional conflict. [18]

Complex regulations that govern maternity leave benefits and the need to navigate these regulations can also interfere with decision-making. FMLA applies only to employees who work at companies with 50 or more employees and who have worked for at least 1,250 hours during the year preceding childbirth. Consequently, only about 20% of new mothers and 50% of all mothers are covered by FMLA. [19,20] State laws vary in coverage of workers in government and small companies and in the generosity of leave. California is one of five (out of 50) U.S. states that offer mothers paid prenatal and postnatal leave through temporary disability insurance (State Disability Insurance (SDI) in California). Funded through employee contributions, SDI provides partial pay up to 4 weeks prior to delivery and 6 to 8 weeks after childbirth with an extension for up to an additional 6 weeks postpartum through the Paid Family Leave program (PFL). SDI is not job-protected leave, although in many cases leave-takers get additional protections under FMLA. For mothers covered by these laws, job-protected (unpaid) and paid leave must be taken concurrently. Employees also must coordinate sick pay and vacation pay from the employer with pay from the state through SDI and PFL. While some companies voluntarily provide paid time off for maternity leave or offer the opportunity to purchase maternity leave coverage from insurance providers, these practices are limited. Evidence shows that public awareness of SDI and PFL programs in California is low, especially among low income, minority and young respondents,[19,21,22] suggesting need for education and outreach to promote maternity leave uptake. [23]

Although some decision-making tools that promote awareness of maternity leave options are available online, we could not find any that covered information about California-specific maternity leave laws or the health benefits of taking leave that have been formally evaluated. Responding to this gap, the objective of our project was to develop an easy-to-use, evidence-based educational resource and evaluate whether it raised awareness about maternity leave and mobilized users to plan and take leave. The tool was developed through a cooperative agreement between University of California, Berkeley’s School of Public Health and the March of Dimes (a national non-profit organization that promotes healthy birth outcomes), California Chapter. The intent was to integrate this tool into national worksite wellness programs and resources offered by the March of Dimes. In this paper we 1) describe the participatory development of the maternity leave educational tool; 2) present the results of a pilot evaluation of the tool with pregnant women using a low-exposure, low-cost intervention intended to elicit interest in reading the tool and facilitating decisions about prenatal and postnatal leave; and 3) discuss challenges and recommendations for future dissemination and evaluation of the tool.

## Materials and Methods

### Five Steps Towards the Development of the Maternity Leave Educational Tools (Fig 1)

**Fig 1:**
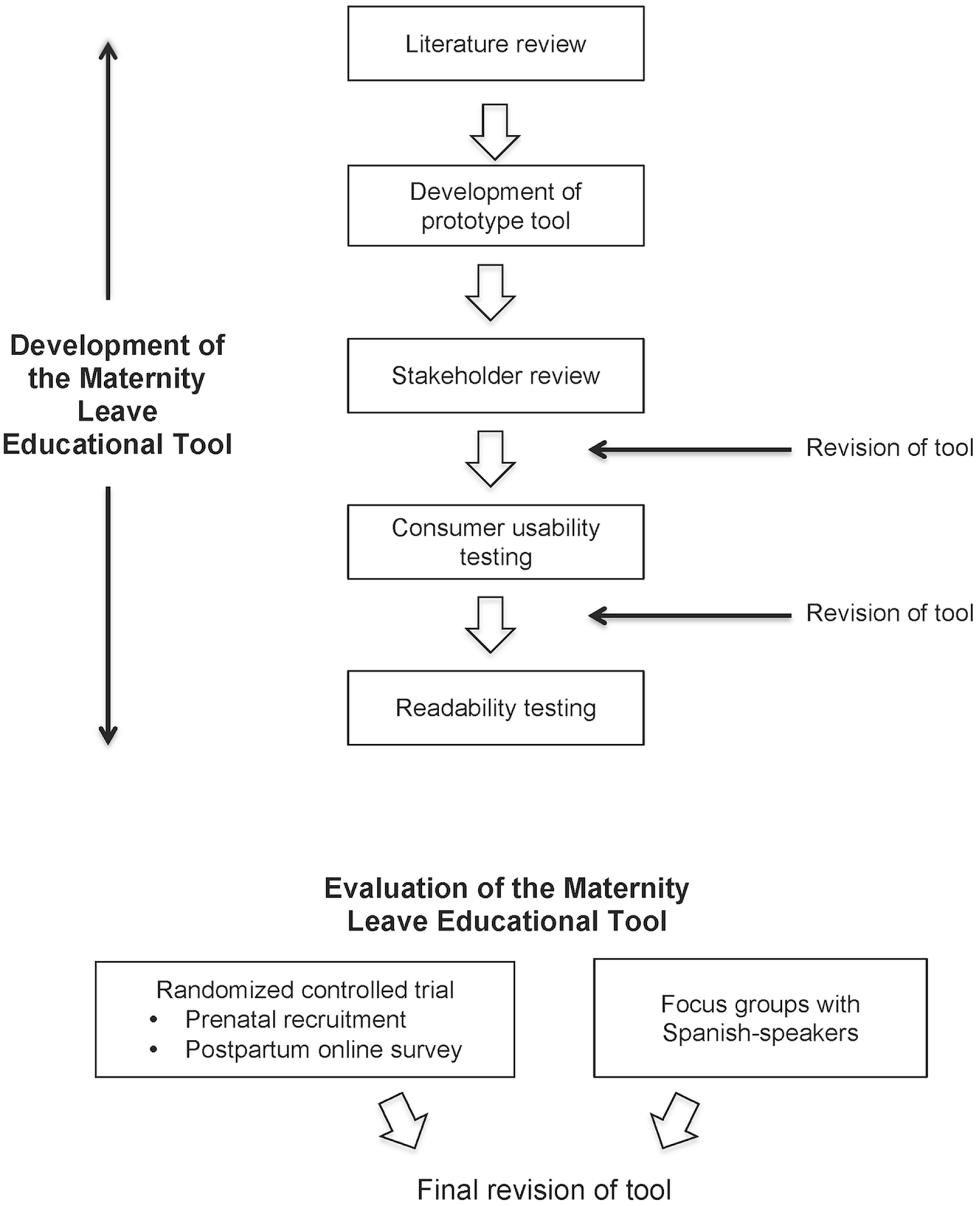
Development and Evaluation of the Maternity Leave Tool.

#### Literature review and content of tool

We reviewed the literature about the health impact of taking prenatal and postnatal leave on both mother and child. We also researched the California paid leave programs and laws protecting women’s jobs during maternity leave. Based on this research, we drafted a one-page (double-sided) maternity leave educational tool (the ‘tool’). Content included the health benefits of taking prenatal and postnatal leave for mother and baby, other considerations when deciding length of leave, information about California paid leave programs and laws, questions to ask one’s employer, and phone numbers and websites for more information.

#### Unformatted tool

We drafted the prototype guided by the following theoretical frameworks and according to health literacy and clear communication principles. The Ottawa Decision Support Framework is an evidence-based, transdisciplinary framework that can be used to help people deliberate about and make informed decisions in a non-directive way that is consistent with their own values. [17] The Transtheoretical Model of Health Behavior identifies decisional stages needed to make behavioral changes. [24] Social semiotics models help explain how people interpret the meaning of communications, and underscore that health messages must be understandable, engaging and motivating to users. [25] Action research and design science models emphasize the importance of using iterative, user-centered development and testing of resources. [26–28] Guidance about developing and testing health communication recommends that health information adhere to health literacy and “clear communication” principles. [29,30] Principles include having text written at or below the average reading level of the intended users and using formats that are easy-to-read, such as having adequate “white space” around text, sufficiently large font sizes, bulleted lists and relevant graphics. In addition, information that is more comprehensible and actionable should emphasize what people can do, rather than just what they should know and provide information about where they can get help. A second important recommendation is that materials should be created and tested with the participation of the intended beneficiaries and relevant stakeholders. [31,32] Such participatory design processes include engaging users and stakeholders to identify problems about understanding and acting on health issues, and to work with experts to co-develop and test information that meets their needs. Participatory, or “user-centered” design processes usually require several rounds of developing and refining prototypes before a testable version is produced. [27] Health communication materials created with participatory design and with health literacy principles have shown significantly improved decisional outcomes. [33–36]

#### Stakeholder review

Seven stakeholders with expertise related to maternity leave, representing obstetrics, law, social work, human resources, and public health, provided feedback on the content of the unformatted tool. We made several iterative revisions to the prototype tool based on their feedback to ensure that all the information in the tool was presented accurately.

#### Consumer usability testing

**Fig 2:**
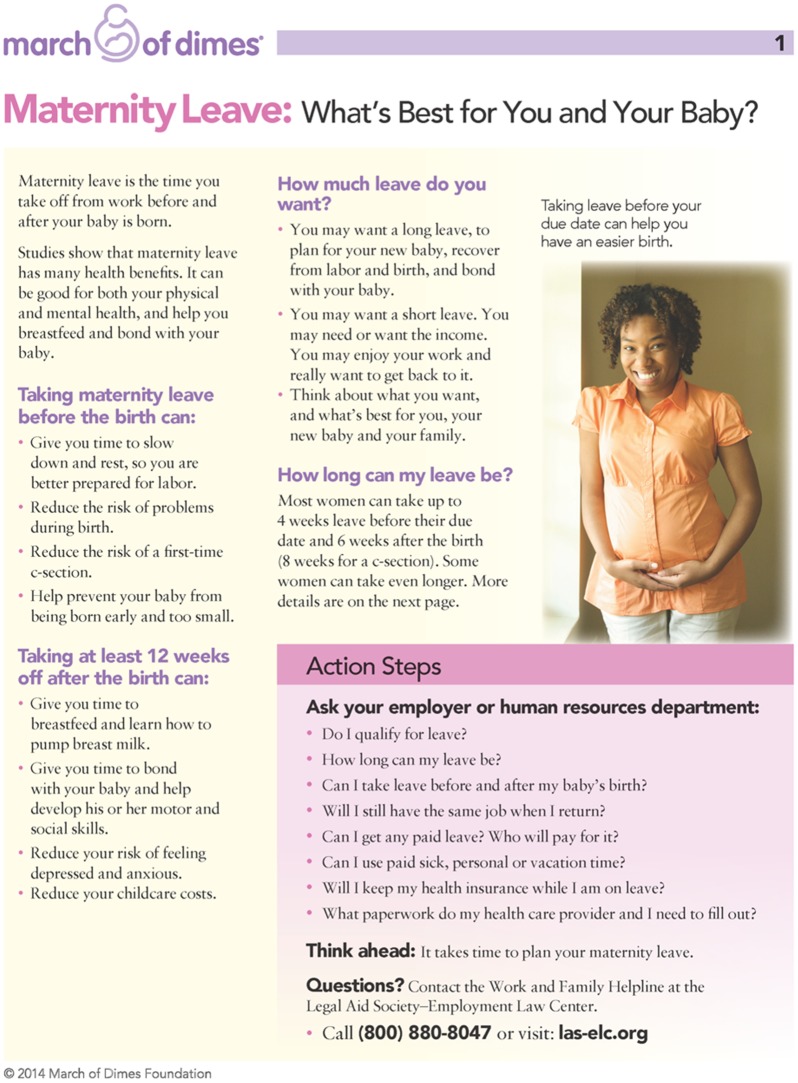
Maternity Leave Educational Tool.

We produced a first draft of the formatted tool, adhering to health literacy and clear communication principles. We then conducted two rounds of in-person usability testing with pregnant, working women (n = 7), intentionally including some women with average-to-low educational levels. The usability testing consisted of semi-structured, one-on-one, in-depth interviews with, and observations of, participants using the draft tool. These sessions queried participants about the comprehensibility and other features of the tool, prompted them to perform tasks using the tool, and asked for suggestions to improve it. We revised the tool after each round of usability testing based on consumer feedback and in consultation with our team’s health communication experts. (See Fig 2 for image of final version of tool.)

(The tool is available at: marchofdimes.com/ca/maternityleave.)

#### Readability testing

The tool was tested for readability with Readability Plus software (Readability Calculations. Dallas, TX: Micro Power & Light Co. 2005). Because the average American is estimated to read between the 7^th^-9^th^ grade level, [37–39] the readability scores met our goal for the tool.

<Editor: Small photo of the tool (Fig 2) should be put about here>

### Evaluation of the Maternity Leave Educational Tool

#### Pilot evaluation

We tested the short-term efficacy of the tool in an initial pilot evaluation, using a design-science evaluation approach that has been successfully applied in engineering, computer science and other socio-technical fields. The first phase of evaluation was intended to test one or more prototypes in what is called a “test and evaluate loop” to allow for additional revisions before launching a large-scale study. [27]

We conducted a randomized controlled trial (Fig 1) administered electronically to pregnant, working mothers residing in the San Francisco Bay Area between June and November 2012. Intervention participants received the tool as a PDF during pregnancy while controls did not receive any educational tools. Approximately one month after their due date, participants were invited to complete an online survey. Participants received no additional educational support, counseling or coaching.

#### Recruitment and screening

We recruited study participants through obstetric offices and flyers posted in various community settings in the Bay Area, including at Women, Infants and Children program (WIC) offices, children’s clothing stores, public libraries, Head Start programs, childcare referral agencies, prenatal education centers, nonprofits that provide family services, farmer’s markets, cafes, and laundromats. We also recruited online through parenting listservs, parenting websites (using paid advertisements), craigslist.org, and Facebook. Potential participants were directed to a website to be screened, consented and enrolled in the study.

Potential participants were screened through an online survey. Only participants who were 24 to 31 weeks pregnant, currently working (and not already on maternity leave), 18 years or older, and living in California were invited to participate in the study. Participants completed an online consent form, and provided their delivery due date and contact information. Eligible participants who enrolled in the study online were randomly assigned to either the intervention or control group by the online Qualtrics survey software (Qualtrics, Provo, UT).

#### Online survey

All participants were re-contacted by email one month after their due date and prompted to take the postpartum survey. We called participants by phone if they did not complete the online survey after three e-mail reminders and sent them additional e-mails.

Survey questions asked participants about their socio-demographic and work characteristics, uptake of prenatal leave and/or postnatal leave, and knowledge about maternity leave. Intervention group participants were asked additional questions about their use of the maternity leave educational tool. We first asked if they recalled getting and reading the tool, and how many times they read it. We asked participants who reported reading the tool their perceptions of several features of the tool:

- how easy it was to understand the tool;
- how useful it was and the most useful thing;
- if they used the tool to find answers to a question they had and if the tool answered that question;
- if they learned something new and what was learned about maternity leave
- if the tool helped them plan their maternity leave;
- if they changed their maternity leave plans based on the tool;
- if they brought the tool to a meeting with their doctor or employer; and
- if they called any of the phone numbers or visited websites listed on the tool.

The University of California, Berkeley, institutional review board (the Committee for the Protection of Human Subjects) approved the study protocol and required participants to provide online consent for the survey.

#### Sample

Of the 660 potential participants screened through an online screening questionnaire, 431 met the inclusion criteria. We noted that 265 offered invalid information (e.g. nonsensical names, e.g. ‘fjkdslfjksdj’, had the same IP address or same email address as other participants, or provided a due date that did not agree with the number of weeks they reported being pregnant) and were excluded from the study. The remaining 166 participants were invited to take the postpartum survey, out of which 155 participants completed the postpartum survey (n = 77 controls and n = 78 intervention participants).

#### Data analysis

Descriptive statistics compared intervention participants who read the tool, intervention participants who did not read the tool, and control arm participants on demographic characteristics. We performed t-tests for continuous variables and chi square tests for categorical variables, except for those with low expected cell counts, where we used Fisher’s exact test, to test statistical significance. In addition, we estimated frequencies of use of the maternity leave tool and of its perceived value among participants who read the tool.

To test whether participants in the intervention group, who received the tool, differed in their maternity leave arrangements from participants who did not receive the tool (control arm), we compared the proportion of participants who took any prenatal leave and the proportion who took more than a week of prenatal leave using a chi square or Fisher’s exact test. We further restricted this analysis to women who reported utilizing the tool to plan their leaves. Women who delivered at ≤37 weeks gestation were excluded from this analysis due to their higher likelihood of having medical complications requiring disability leave or going into labor before their planned prenatal leave could begin.

We further compared the proportion of participants who took any postnatal leave and the proportion that took at least eight weeks of postnatal leave by control vs. intervention group. For the latter analysis, we excluded 1) participants who had no plans to return to work in the next year, and 2) participants who took the survey prior to eight weeks postpartum but who had not yet returned to work. We also performed sensitivity analyses treating the outcomes as continuous variables. All analyses were conducted using SAS software (SAS, v9.2. Cary, NC).

Because 54% of intervention participants reported that they did not read the tool, we conducted a *post-hoc* chi square analysis comparing maternity leave for three groups: controls, intervention participants who read the tool, and intervention participants who did not read the tool. Nine intervention participants who did not report whether or not they read the tool were dropped from the analysis.

## Results

### Demographics

As shown in Table 1, participants were predominantly in their 30s, Caucasian, married, and highly educated; 89% had an undergraduate degree or higher. Most participants were working full time during their pregnancy, and nearly half exceeded a 40-hour workload. The majority (90%) had been at their present job for at least a year, and most (70%) worked for an employer with 50 or more employees (indicating that they might be eligible for FMLA job-protected (unpaid) leave). Nearly all participants entered prenatal care during their first trimester. For over half of participants, the current pregnancy resulted in their first live birth.

**Table 1:** Characteristics of the Study Participants.

	Control group (n = 77)	Intervention group^a^ (n = 69)	
		Did not read tool (n = 32)	Read tool (n = 37)
	*n (%)*	*n (%)*	*n (%)*
Age			
18–29 years	13 (16.9)	2 (6.3)	7 (18.9)^b^
30–39 years	54 (70.2)	24 (75.0)	29 (78.38)
40+ years	10 (13.0)	6 (18.8)	1 (2.7)
Race/ethnicity			
Caucasian	58 (75.3)	20 (62.5)	25 (67.6)
African American^c^	4 (5.2)	0 (0)	4 (10.8)
Latino or Hispanic^c^	6 (7.8)	1 (3.1)	4 (10.8)
Asian	6 (7.8)	11 (34.4)	4 (10.8)^b^
American Indian or Alaskan Native^c^	2 (2.6)	0 (0)	0 (0)
Pacific Islander^c^	1 (1.3)	0 (0)	0 (0)
Other^c^	2 (2.6)	1 (3.1)	1 (2.7)
Education			
Some college or technical school or less	12 (15.6)	1 (3.1)	3 (8.1)
College graduate	15 (19.5)	10 (31.3)	12 (32.4)
Graduate or professional school	50 (64.9)	21 (65.6)	22 (59.5)
Annual household income			
Less than $50,000	10 (13.2)	3 (9.7)	4 (11.1)
$50,000–$74,999	5 (6.6)	3 (9.7)	6 (16.7)
$75,000–$99,999	14 (18.4)	3 (9.7)	6 (16.7)
$100,000–149,999	17 (22.4)	9 (29.0)	9 (25.0)
$150,000 or more	30 (39.5)	13 (41.9)	11 (30.6)
Marital status^c^			
Married	61 (80.3)	29 (90.6)	30 (81.1)
Living with partner	11 (14.5)	2 (6.3)	7 (18.9)
Never married	4 (5.3)	1 (3.1)	0 (0)
Entry into prenatal care^c^			
First trimester	76 (98.7)	31 (100)	36 (97.3)
Second trimester	1 (1.3)	0 (0)	1 (2.7)
Weeks pregnant when enrolled in study, mean (SD)	27.8 (2.6)	28.8 (2.4)	27.9 (2.3)
Weeks postpartum when took survey, mean (SD)	10.1 (7.2)	12.1 (8.6)	13.4 (9.1)^d^
Number of pregnancies			
1	32 (41.6)	13 (40.6)	14 (37.8)
2–3	32 (41.6)	16 (50.0)	20 (54.1)
4 or more	13 (16.9)	3 (9.4)	3 (8.1)
Hours worked per week when 6–7 months pregnant^c^			
1–20 hours	4 (5.3)	1 (3.1)	5 (13.5)^b^
21–30 hours	10 (13.2)	2 (6.3)	7 (18.9)
31–40 hours	39 (51.3)	20 (62.5)	10 (27.0)
More than 40 hours	23 (30.3)	9 (28.1)	15 (40.5)
Length of time working at job^c^			
Less than 1 year	9 (11.7)	4 (12.5)	2 (5.4)
More than 1 year	68 (88.3)	28 (87.5)	35 (94.6)
Number of people working for employer			
Fewer than 5	9 (11.7)	3 (9.4)	5 (13.5)
Between 5 and 49	12 (15.6)	9 (28.1)	6 (16.2)
50 or more	56 (72.7)	20 (62.5)	26 (70.3)

^a^ Does not include nine intervention participants who did not report whether they read the tool or not.

^b^ Statistically significant difference (p<0.05) between 'read tool' intervention group and 'did not read tool' intervention group; analyzed using chi square test for categorical variables and t-test for continuous variables except where otherwise noted.

^c^ Analyzed using Fisher's exact test.

^d^ Statistically significant difference (p<0.05) between 'read tool' group and control group; analyzed using chi square test for categorical variables and t-test for continuous variables except where otherwise noted.

Among participants randomized to the intervention arm, 54% reported having read the tool. Intervention group participants who read the tool and control participants did not differ on any demographic characteristics except in number of weeks postpartum when they took the survey (Table 1; p = 0.04). Among the intervention participants, those who read the tool were younger (p<0.05), non-Asian (p = 0.02) and more likely to be working part-time (p = 0.02) than those who did not read the tool.

### Educational tool use and value

Among participants who read the tool, most looked at it 1–2 times (70%), and 19% reported looking at it 3–4 times (Table 2). Two-thirds of participants felt that it was very easy to understand and 30% thought it was somewhat easy to understand. Although almost two-thirds considered that they knew somewhat or a lot about maternity leave prior to reading the tool, the majority reported that the tool was somewhat useful (62%) or even very useful (30%) and 65% stated that they had learned something new from the tool. Participants reported that the clear and concise description of the different types of leave available was the most useful aspect of the tool. Several women valued the information about how much time off (pre- and postnatal) they could take using each type of leave—namely paid leave and job—protected (unpaid) leave and a number appreciated having all of the California state laws in one place, including contact numbers to call for further information. A few also mentioned that the information on health benefits of taking maternity leave was useful in helping them plan their maternity leave.

**Table 2:** Educational Tool Use Questions (Intervention Group Participants Who Reported Reading Tool Only).

	Read tool (n = 37)
	*n (%)*
**Use and perception of tool**	
Number of times looked at tool	
1–2 times	26 (70.3)
3–4 times	7 (18.9)
5 or more times	3 (8.1)
Don't know	1 (2.7)
Tool easy to understand?	
Very easy to understand	25 (67.6)
Somewhat easy to understand	11 (29.7)
Somewhat hard to understand	1 (2.7)
Very hard to understand	0 (0)
Usefulness of the tool	
Very useful	11 (29.7)
Somewhat useful	23 (62.2)
Not very useful	2 (5.4)
Not at all useful	1 (2.7)
Tool answered questions	
Yes	8 (61.5)
Partially answered	5 (38.5)
No	0 (0)
Learned something new from the tool	24 (64.9)
BEFORE received tool, how much knew about maternity leave?	
A lot	11 (29.7)
Some	12 (32.4)
A little	13 (35.1)
Nothing	1 (2.7)
**Tool helped to deliberate about leave-taking**	
Referred to the tool to find answers to questions	13 (35.1)
Called any phone numbers or visited any websites listed on tool	14 (37.8)
Took the tool to meeting with doctor or human resources representative	4 (10.8)
**Tool helped to make a decision**	
Tool helped to plan maternity leave	18 (48.7)
Changed maternity leave plans based on information from tool	5 (13.5)

### Tool as a deliberation aid

One-third of participants reported that the tool motivated them to deliberate about maternity leave by finding answers to questions, calling phone numbers or checking websites listed in the tool, and/or by taking the tool to discuss with doctors or human resource representatives (Table 2). Those who used it to deliberate on whether to take leave reported that the tool educated them on how much paid time off they could receive, which in turn, helped them to decide how much maternity leave they could afford to take. Others said the tool helped them in negotiating with their employer; they learned the appropriate terms, questions they should ask, and how much leave they were eligible for.

### Tool as a decision-making aid

The short-term efficacy of the tool was assessed by two questions: 1) Did the tool help you plan your maternity leave? and 2) Did you change your maternity leave plans based on the information that the tool provided? Notably, nearly half of the participants said the tool had helped them plan their maternity leave and 14% reported that they had changed their leave plans based on the information they learned from the tool (Table 2).

### Prenatal and postnatal leave uptake

Seventy-eight percent of women who delivered at ≥ 37 weeks gestation took some amount of prenatal leave. The mean length of leave was 3 weeks. A greater proportion of intervention participants (82%) took some prenatal leave compared with controls (74%) (p = 0.41) and among prenatal leave takers, a larger proportion of intervention participants (71%) took more than a week of prenatal leave compared with controls (64%) (p = 0.47) (Table 3).

**Table 3:** Prenatal and Postnatal Leave Taken.

	Control group (n = 77)	Intervention group (n = 78)	p value
**Prenatal leave** ^a^	*n (%)*	*n (%)*	
Took any prenatal leave (n = 144)	52 (74.3)	59 (79.7)	0.44
Took >1 week prenatal leave (n = 144)	45 (64.3)	52 (70.3)	0.44
Weeks of prenatal leave^b^, mean (SD) (n = 111)	3.0 (1.9)	3.2 (2.2)	0.54
**Postnatal leave**			
Took any postnatal leave (n = 155)	76 (98.7)	77 (98.7)	1
Took 8+ weeks postnatal leave (n = 70)^c^	29 (87.9)	31 (83.8)	0.74
Weeks of postnatal leave^d^, mean (SD) (n = 50)	12.2 (5.7)	13.3 (6.1)	0.54

^a^ Only includes participants who delivered at 37 weeks gestation or later.

^b^ Only asked of participants who reported taking prenatal leave and who delivered at 37 weeks gestation or later.

^c^ Excludes participants who had no plans to return to work in the next year and participants who took the survey prior to eight weeks postpartum but who had not yet returned to work.

^d^ Only includes participants who had already returned to work and who provided a date of return to work.

Notably, although we found no differences in prenatal leave uptake across intervention participants who read the tool, intervention participants who did not read the tool and controls, among participants who reported that the tool had helped them plan their leaves, 89% took more than one week of prenatal leave, a proportion that was significantly higher than among controls (64%, p = 0.05) (data not shown).

All but two participants reported taking some postnatal leave. Among participants who had already returned to work by the time of the survey (mean 12.9 weeks postpartum) (n = 49), the mean length of postnatal leave taken was 12.2 weeks among control participants and 13.4 weeks among intervention participants (p = 0.52).

When we compared the maternity leave outcomes between the three groups (controls, intervention participants who read the tool, and intervention participants who did not read the tool), we found no evidence of a difference between groups on any of the outcomes—the proportion who took any prenatal leave (p = 0.41), at least a week of prenatal leave (p = 0.59), any postnatal leave (p = 0.63), and at least eight weeks of postnatal leave (p = 0.58).

## Discussion

This study assessed the short-term efficacy of a maternity leave tool designed to raise awareness of maternity leave and encourage pregnant working women in California to plan and take childbirth-related leave. Pilot evaluation findings from the randomized controlled trial show that the tool helped to raise awareness about maternity leave and effectively mobilized participants to take action. Concretely, over half of the online intervention group participants read the tool and almost two-thirds reported learning something new from the tool. Furthermore, for nearly half of the intervention arm participants, the tool helped them plan their leave, nearly 40% reported calling a telephone number listed in the tool to find more information, and 11% took the tool to confer with doctors or human resource representatives. Raising awareness about maternity leave and mobilizing participants to take action are important first steps in a country like the U.S., and even in a progressive state like California, where maternity leave uptake is much lower than many other industrialized countries. In addition, the results of the intervention can be considered positive in the context of evidence about the effects of “low-touch,” mass media, online health communication and considering that the trial enrolled primarily highly educated, affluent participants, many of whom knew about maternity leave prior to receiving the tool.

Results also indicate that both prenatal and postnatal leave uptake and duration were higher overall among intervention than control participants, but did not reach statistical significance, perhaps because the sample was too small. Unlike changes in awareness, changes in maternity leave uptake may be more difficult to achieve given that choices in California are constrained by eligibility laws, informational barriers and economic factors in so far as paid programs only offer up to 55% of wage replacement (www.edd.ca.gov) and there is lack of job protection for employees not covered by FMLA. Alternatively, among the more educated women in our sample, most of whom worked for large employers covered by FMLA, we may have observed a reduced responsiveness to change because these women may have already received information and made decisions about maternity leave.

Nonetheless, the tool did achieve some modest results in improving maternity leave uptake. Among participants who delivered at term and said the tool helped them plan their leave, 89% took more than one week of prenatal leave, a significantly higher proportion than among controls who did not receive the tool. Previous studies have shown benefits of prenatal leave uptake for healthy women who deliver at term, including lower risk of primary cesareans and other obstetric complications [4,6] and lower risk of preterm delivery among women employed in physically strenuous jobs. [1,40] Because in California paid prenatal leave cannot be transferred to the postpartum period, taking leave before the child is born does not limit opportunities for maternal-infant bonding after birth.

The study had several limitations. The tool is designed to raise awareness and utilization of maternity leave in California and its value to other industrialized countries where maternity leave is paid and widely accepted as the norm, may be limited. Application in other states within the US will require adaptation to state-specific laws. Our sample size for evaluating the tool was small. We now estimate that in order to detect a seven-day difference in postpartum leave between intervention and control group (as observed in this pilot), a larger study of approximately 1000 to 1300 women will be required. We found it very difficult to engage health care providers as partners in recruiting participants and relied on various community sources and online venues for recruitment. Our screening procedures were not initially able to detect false online enrollment information provided by enrollees, requiring us to drop them from the online study after enrollment once reliability checks could be performed. As a result, the online survey sample size was smaller than intended.

Although we used health literacy principles to ensure that questions were easy to understand and answer, the online survey instrument was not validated. Survey participants tended to be well-educated, English speakers and not fully representative of Californian pregnant, working women. Although we recruited a diverse group of women, online participants who read the tool tended to be young, non-Asian and part-time workers. Future evaluations will require assessing the impact of the tool among full-time workers and non-affluent, multi-racial, and non-English-speaking, working pregnant women. Furthermore, it is possible that more intervention participants would have used the tool in planning the length and timing of their leave if it had been provided earlier.

A further issue, supported by the findings of health communication studies, is that having information from a trusted source is often important when people need to understand and make decisions about health risks and benefits. [41] A stand-alone website, like the one used in this study, would not meet that criterion. Therefore, providing this information in a “mediated” way—given out by a trusted health care provider, an employer, a human resource representative, or some other way that connects the recipient to a person or organization—might encourage more women to read it and to trust the information that they read. [22] The tool could be posted on websites where pregnant women might seek out information about pregnancy and maternity leave, e.g., the March of Dimes website or a legal aid center website. The tool could also be incorporated into larger scale information campaigns for pregnant women, such as the California First 5 Kit for New Parents (educational resources disseminated to hundreds of thousands of expectant and new parents each year in California). [41] Furthermore, the tool could be incorporated into human resources materials for employees, including a company’s internal website or wellness program for employees. Future studies will need to evaluate the impact of the tool on employer readiness and employment-related behaviors.

In summary, this maternity leave tool showed promising results for use, reported learning and planning leave among pregnant women who used it during their late second and third trimesters. By developing and evaluating a California-specific maternity leave tool that provides information on laws and health benefits of taking leave, this study succeeded in filling a gap in the health educational materials currently available to pregnant, working women.

Based on these study findings, we recommend that the tool be distributed to pregnant women earlier, perhaps as early as their first trimester and that it be distributed in a mediated way through a health or work organization to which women are connected or embedded in other motivating materials about pregnancy. We also recommend that this first phase evaluation be followed up with research to test the tool using mediated distribution among a larger sample of pregnant working women. Reducing informational barriers and allowing women to more easily navigate the complicated regulations that govern maternity leave in California, might help women access a benefit that is far less available than it is for working women in other industrialized countries.
